# Young Barley (*Hordeum vulgare* L.) Preparations: From Phytochemical Complexity to Clinical Relevance

**DOI:** 10.3390/molecules31122190

**Published:** 2026-06-22

**Authors:** Wojciech Rzeski, Weronika Rzeska

**Affiliations:** 1 Department of Functional Anatomy and Cytobiology, Institute of Biological Sciences, Maria Curie-Skłodowska University, Akademicka 19, 20-033 Lublin, Poland; 2 Doctoral School of Medical Sciences, Medical University of Lublin, 20-093 Lublin, Poland; weronikarzeska@gmail.com

**Keywords:** young barley, barley grass, *Hordeum vulgare*, saponarin, lutonarin, functional plant food, antioxidant activity, phytochemical variability, narrative review

## Abstract

Young barley, derived from the early vegetative stage of *Hordeum vulgare* L., constitutes a plant-based functional ingredient whose phytochemical profile differs markedly from that of mature grain. Two principal commercial forms exist—dried grass powder and juice-derived products—differing in matrix composition and bioactive compound concentration. This narrative review critically evaluates the current knowledge on the phytochemical composition, biological activity, and translational relevance of young barley preparations considered as a functional plant food. The phytochemical spectrum is dominated by C-glycosyl flavones, particularly saponarin and lutonarin, alongside phenolic acids, chlorophylls, enzymatic antioxidants, vitamins, and minerals. Experimental evidence implicates the modulation of redox homeostasis, inflammatory signaling, and metabolic regulators as the primary biological mechanisms. In vitro studies additionally demonstrate antiproliferative activity in human cancer cell lines and immunomodulatory properties mediated by polysaccharide-rich fractions, extending the biological profile of young barley beyond classical antioxidant activity. Although preclinical models consistently demonstrate antioxidant and metabolic effects, high experimental doses and limited preparation standardization restrict the direct extrapolation to human supplementation contexts. Available clinical trials suggest modest improvements in selected lipid, glycemic, and oxidative stress markers; yet, most are small in scale and brief in duration. Agronomic variables including fertilization strategy and soil composition represent additional, underappreciated sources of phytochemical variability and safety concern. Overall, the current evidence supports the biological plausibility of young barley as a functional plant food; yet, the clinical data remain preliminary. Future research should prioritize preparation standardization, dose–response characterization, and agronomic transparency to strengthen translational reliability. In conclusion, young barley preparations represent a biologically plausible functional plant food ingredient with preliminary clinical support, pending confirmation from adequately powered, standardised randomised controlled trials.

## 1. Introduction

Young barley, derived from the early vegetative stage of *Hordeum vulgare* L., has attracted substantial scientific interest as a plant-based functional ingredient bridging nutrition, phytochemistry, and preventive health. Unlike mature barley grain, which is primarily valued for its carbohydrate and fiber content, young barley harvested at the leaf stage contains a distinct spectrum of secondary metabolites, photosynthetic pigments, enzymatic antioxidants, and micronutrients [1,2,3,4]. This compositional difference underlies its classification as a cereal grass product and a functional plant food rather than a conventional cereal.

Cereal grasses feature in diverse traditional dietary practices; yet, rigorous scientific inquiry into young barley preparations has expanded substantially over the past two decades [2,4,5,6]. The rapid market growth for so-called “green superfood” supplements has been accompanied by wide-ranging claims regarding antioxidant capacity, metabolic support, and general well-being. Validating such claims scientifically demands a clear separation between mechanistic plausibility and clinically demonstrated efficacy, a distinction not consistently maintained in the current literature.

Young barley is commercially available in two principal forms: powdered grass produced by drying and milling the leaves, and juice-derived products obtained by pressing fresh leaf sap followed by stabilization through freeze-drying. These formulations diverge in matrix composition, processing history, and potentially in phytochemical concentration and bioavailability [7,8,9]. Powdered preparations retain structural fiber components, while juice-derived products may be enriched in soluble compounds and labile antioxidants. Despite this distinction, direct comparative evaluations of the two formulations remain sparse in the published literature.

The phytochemical profile of young barley is defined by the presence of C-glycosyl flavones, principally saponarin and lutonarin, together with phenolic acids, chlorophylls, carotenoids, enzymatic antioxidants, vitamins, and minerals [10,11,12,13,14,15]. The constituent concentrations are sensitive to cultivar, harvest timing, agronomic practice, and processing method [12,13,14], introducing a layer of compositional complexity that complicates the interpretation of experimental and clinical data.

Given these considerations, the present review aims to: (i) summarize the phytochemical composition of young barley preparations; (ii) evaluate the mechanistic evidence from in vitro and in vivo studies; (iii) assess the available clinical data with attention to methodological limitations; (iv) discuss regulatory and quality control aspects; and (v) examine the impact of cultivation practices on phytochemical variability and safety. The review is intended for researchers in food science, phytochemistry, and clinical nutrition, as well as regulators and product developers working with functional plant food ingredients. A comprehensive critical synthesis integrating compositional, mechanistic, clinical, and agronomic perspectives has not previously been available for young barley preparations, representing the primary justification for this work.

This narrative review focuses exclusively on preparations derived from the young vegetative stage of *Hordeum vulgare* L.—encompassing barley leaf, barley grass, and juice-derived products. Literature was identified through searches of PubMed, Scopus, and Web of Science using the terms “young barley,” “barley grass,” and “Hordeum vulgare,” without restriction on publication year. Studies on mature barley grain, other cereals, or barley sprouts were included only where they provided a mechanistic context directly relevant to the phytochemicals characteristic of young barley preparations. No formal meta-analytic synthesis was performed. In the context of this review, “young barley” refers to plants harvested at the leaf stage, conventionally 7–15 days post-germination and prior to jointing, at which point C-glycosyl flavone concentrations are at or near their peak.

## 2. Phytochemical Composition of Young Barley

Young barley harvested at the early vegetative stage exhibits a phytochemical profile that diverges substantially from that of mature grain. Its compositional spectrum encompasses polyphenolic compounds, photosynthetic pigments, enzymatic antioxidants, vitamins, and minerals. The relative abundance of these constituents is governed by harvest timing, cultivation conditions, and processing method, collectively accounting for the variability observed across commercial preparations [10,12,15,16]. A merged overview of major bioactive compound classes, their concentration ranges, stability characteristics, and biological relevance is presented in Table 1.

### 2.1. Polyphenolic Profile and C-Glycosyl Flavones

Among the secondary metabolites of young barley, C-glycosyl flavones stand out as the most characteristic and extensively studied class of bioactive compounds. Saponarin (apigenin-6-C-glucosyl-7-O-glucoside) and lutonarin (luteolin-6-C-glucosyl-7-O-glucoside) are regarded as the principal representatives [15,17,18,19]. Chromatographic quantification has revealed considerable variability in flavonoid concentration depending on cultivar, harvest stage, and extraction procedure, including the influence of light quality on saponarin accumulation [11,23]. Given this variability, saponarin and lutonarin have been proposed as the most suitable marker compounds for the analytical standardization of young barley preparations [15,17,19]. Reporting the quantitative concentrations of these flavones should be considered a minimum requirement for preparation characterization in both experimental and clinical studies.

In addition to C-glycosyl flavones, phenolic acids such as ferulic, p-coumaric, and caffeic acids contribute to the total phenolic content of young barley [12,19]. Although phenolic acids are often present at lower concentrations than flavones, they may enhance the overall antioxidant capacity through additive or complementary interactions [24,25]. A structurally relevant property of C-glycosyl flavones is their enhanced resistance to hydrolysis relative to their O-glycosylated counterparts, which confers greater gastrointestinal stability and facilitates transit to the distal intestine where microbial biotransformation may occur [26,27].

### 2.2. Photosynthetic Pigments, Enzymatic Antioxidants, and Micronutrients

Young barley leaves accumulate substantial quantities of photosynthetic pigments, with chlorophyll a and chlorophyll b reaching peak concentrations during early vegetative growth [8,10,12]. Both chlorophyll derivatives and carotenoids contribute to the redox-active capacity and are thought to participate in the attenuation of oxidative processes [28]. Enzymatic antioxidants, particularly superoxide dismutase (SOD), have been reported in young barley preparations [20]. However, enzymatic activity is highly sensitive to processing conditions and may decline substantially during thermal drying [13]. Freeze-drying techniques appear to preserve enzymatic integrity more effectively than conventional air-drying methods [13]. The contribution of enzymatic antioxidants to systemic activity following oral supplementation remains uncertain, as protein digestion in the gastrointestinal tract may limit the bioavailability of intact enzymes [20]. The proposed mechanisms by which photosynthetic pigments, enzymatic antioxidants, and associated micronutrients may confer biological benefits are discussed in detail in Section 3.

Beyond pigments and enzymatic antioxidants, young barley preparations contain vitamins—including vitamin C, vitamin E, and provitamin A carotenoids—alongside essential minerals such as iron, magnesium, zinc, and potassium [1]. While these micronutrients contribute to the overall nutritional value, their concentrations vary depending on soil composition and fertilization practices [1]. The mineral content may also reflect environmental exposure, underscoring the importance of soil quality assessment and contaminant screening [22].

## 3. Molecular Mechanisms of Action

The biological activity of young barley preparations is thought to emerge from the coordinated modulation of redox homeostasis, inflammatory signaling, and metabolic regulation. A critical caveat applies throughout this section: the vast majority of mechanistic insights derive from in vitro systems or animal models employing concentrated extracts. Mechanistic conclusions should therefore be regarded as biologically plausible hypotheses rather than confirmed accounts of pathway engagement at physiologically relevant dietary doses.

Flavonoid-rich fractions from young barley have been linked to the activation of the nuclear factor erythroid 2-related factor 2 (Nrf2) pathway, a master regulator of cellular antioxidant responses [29,30], with the downstream induction of cytoprotective enzymes such as heme oxygenase-1 and NAD(P)H quinone oxidoreductase 1 [31,32]. This pattern implies a shift toward enhanced endogenous resilience to oxidative challenge rather than merely transient radical scavenging. The anti-inflammatory activity observed in cellular systems is commonly attributed to the attenuation of nuclear factor kappa B (NF-κB) signaling, with reduced expression of downstream inflammatory mediators including inducible nitric oxide synthase (iNOS) and cyclooxygenase-2 (COX-2) in macrophage models [33,34,35,36,37]; at the isolated compound level, lutonarin (20–60 μM) and saponarin (80 μM) dose-dependently suppressed NF-κB activation and pro-inflammatory mediator expression (IL-6, TNF-α, COX-2, iNOS) in RAW 264.7 macrophages [34,35]. Recent in vitro and in silico studies have further confirmed the anti-inflammatory potential of isolated saponarin, reinforcing its role as a key bioactive constituent of young barley preparations [37].

Metabolic regulation represents a third mechanistic axis. AMP-activated protein kinase (AMPK) is a central energy sensor integrating glucose and lipid metabolism [38], and preclinical studies on young barley preparations have reported lipid- and glucose-lowering effects in animal models [39,40], consistent with modulation of metabolic regulatory pathways including AMPK; however, direct measurement of AMPK phosphorylation in these studies was not performed and mechanistic interpretation remains inferential.

Additional mechanistic observations have been reported in cancer cell models. Young barley extracts have been shown to inhibit proliferation and promote apoptotic signaling in human colon and lung cancer cell lines [41,42], and synergistic antiproliferative effects have been demonstrated when young barley extracts are combined with chlorella in breast and colon cancer models [43,44]. Notably, polysaccharide-rich fractions of young barley, particularly fructooligosaccharides, have been shown to enhance natural killer (NK) cell cytotoxicity against human colon cancer cells, pointing to an immunomodulatory dimension of activity that extends beyond the flavonoid fraction [45].

Finally, the intestinal microbiota may represent an underappreciated mediator of systemic effects. C-glycosyl flavones show relative resistance to upper gastrointestinal hydrolysis and may reach the colon, where microbial transformation can yield metabolites with distinct bioavailability and activity profiles [26,27,46]. Eubacterium cellulosolvens has been identified as a principal bacterial species capable of cleaving C- and O-glucosidic linkages in dietary flavonoids, releasing the corresponding aglycones apigenin and luteolin [27]. These aglycones are subsequently subject to further ring-fission and reductive reactions, generating phenolic acids that may exert systemic anti-inflammatory effects at concentrations achievable through dietary exposure [46].

## 4. Evidence from In Vitro and In Vivo Studies

In vitro investigations represent the most extensive body of experimental evidence on the biological activity of young barley preparations. Both grass-powder-derived and juice-derived extracts demonstrate measurable antioxidant activity using standard chemical assays including 2,2-diphenyl-1-picrylhydrazyl (DPPH), 2,2′-azino-bis(3-ethylbenzothiazoline-6-sulfonic acid) (ABTS), ferric reducing antioxidant power (FRAP), and oxygen radical absorbance capacity (ORAC) [47], generally correlating with total phenolic content [48]. Quantitative profiling has shown that barley sprout powder can contain approximately 8.1 mg saponarin per gram dry weight, with the antioxidant capacity following the order FRAP > DPPH > ABTS [49]. Comprehensive reviews of the molecular mechanisms of barley functional ingredients further support the multi-target biological profile of young barley preparations [50]. Anti-inflammatory activity has been extensively investigated in lipopolysaccharide (LPS)-stimulated macrophage models, with reduced nitric oxide (NO) production and decreased secretion of pro-inflammatory cytokines including tumor necrosis factor-alpha (TNF-α) and interleukin-6 (IL-6) [33,37,50].

Antiproliferative and cytoprotective effects have been demonstrated in multiple human cancer cell lines. Aqueous and juice extracts of young *Hordeum vulgare* inhibited proliferation in HT-29 colon and A549 lung cancer cell lines [42], and chemopreventive activity in colon cancer models was further corroborated by studies employing different extraction methods [41]. Synergistic antiproliferative effects were observed when young barley extracts were combined with chlorella [43,44], and the immunomodulatory potential of polysaccharide-rich young barley water extracts was demonstrated through enhancement of NK cell activity against colon cancer cells [45]. Young barley grass dietary supplement extracts also conferred protection against UV-induced oxidative injury in human dermal fibroblasts [9].

Animal model studies using young barley leaf, grass juice, and related preparations have reported directionally consistent metabolic benefits. Early investigations demonstrated that green juice from young barley leaves reduced dietarily induced hypercholesterolaemia in rats [51]. Barley leaf essence similarly attenuated blood cholesterol, triglycerides, and atherosclerotic markers in a rabbit model of cardiovascular disease [39]. More recently, barley grass juice was shown to reduce body weight, BMI, and improve lipid profile in high-fat diet-induced obese rats, with associated reductions in hepatic markers and modulation of PPAR-γ expression [40]. The doses administered in animal experiments frequently correspond to high human-equivalent intakes [52], and the magnitude of effects may not directly reflect achievable outcomes in typical supplementation contexts. A consolidated overview of representative in vitro, in vivo, and clinical findings is presented in Table 2.

A compositionally relevant distinction exists between whole grass powder and juice-derived preparations. The freeze-drying of expressed juice preferentially retains heat-sensitive enzymatic antioxidants and soluble low-molecular-weight phenolics, whereas hot-air drying results in greater degradation of total chlorophyll and flavonoids [7,13]. Juice-derived preparations have in some studies shown higher apparent radical-scavenging capacity [7,9].

## 5. Clinical Evidence

Relative to the extensive preclinical literature, the available clinical evidence on young barley preparations is notably sparse. A comprehensive search of the published literature identified only five human intervention studies that used preparations derived exclusively from the young vegetative stage of *Hordeum vulgare* L.—encompassing barley leaf extract, barley grass powder, barley leaf powder, and barley green—as the primary intervention. The limited number of trials, their heterogeneous designs, and the diversity of preparations collectively preclude definitive conclusions regarding clinical efficacy. This scarcity of human evidence should be regarded not as a peripheral limitation but as a central finding of this review, underscoring the substantial translational gap between the extensive preclinical activity and verified human outcomes.

Venugopal and Iyer [53] investigated barley grass powder (BGP) supplementation at 1.2 g/day for 60 days in 59 stable T2DM subjects, reporting significant reductions in FBS, HbA1c, total cholesterol, and LDL-C alongside an increase in HDL cholesterol. Although the non-randomized design limits interpretive value, these findings provide preliminary clinical evidence for the cardiometabolic potential of barley grass powder across multiple metabolic endpoints simultaneously (reported reductions in the experimental group: FBS 10.8%, HbA1c 5.2%, total cholesterol (TC) 5.1%, LDL-C 8.2%; HDL-C increase 5.0% [53]).

Takano et al. [54] demonstrated that young barley leaf powder (BLP, 1.8 g/serving) suppressed postprandial blood glucose increments in healthy volunteers with higher baseline glucose responses, attributing the effect to increased digesta viscosity by insoluble barley leaf fiber. This study is notable for identifying a glucose-responder subgroup as a potential target population for young barley supplementation.

Yu et al. [55] reported that young barley leaf extract combined with antioxidant vitamins reduced LDL oxidation and improved free radical scavenging activity in adults with T2DM. In a separate study by the same group, Yu et al. [57] administered 15 g/day young barley leaf extract to 40 hyperlipidemic subjects over four weeks, demonstrating reductions in plasma total cholesterol and LDL-C and an increased lag phase of LDL oxidation, with a more pronounced antioxidative effect in non-smokers. Taken together, these two studies provide consistent evidence that young barley leaf extract reduces both circulating lipids and LDL oxidizability in metabolically at-risk populations.

Most recently, Cui et al. [56] conducted the most methodologically rigorous clinical trial on young barley preparations to date: a randomized controlled trial in 90 subjects with hyperuricemia comparing barley green supplementation plus a balanced diet versus dietary modification alone over three months. The intervention group demonstrated significantly greater reductions in serum uric acid (primary endpoint: *p* = 0.049), alongside improvements in xanthine oxidase activity and body composition. This is also the first clinical study to document the efficacy of young barley in hyperuricemia—a metabolic condition with a plausible mechanistic link to the xanthine oxidase inhibitory properties of barley-derived flavonoids.

Across all five studies, safety profiles were generally favorable, with adverse events limited to mild and transient gastrointestinal discomfort. The available clinical evidence spans glycemic control, lipid metabolism, oxidative stress, and uric acid regulation—a heterogeneous set of endpoints reflecting the multi-target profile of young barley phytochemicals. The principal methodological limitations include: (i) small sample sizes; (ii) short intervention durations (predominantly 4–12 weeks); (iii) inadequate blinding or randomization in most trials; and (iv) insufficient preparation characterization, with few studies reporting quantitative saponarin or lutonarin concentrations. The totality of evidence is preliminary and hypothesis-generating. Adequately powered, double-blind randomized controlled trials with standardized, analytically characterized young barley preparations and pre-specified primary endpoints represent the most pressing unmet need in this field.

## 6. Regulatory and Quality Control Considerations

The rapid global expansion of the young barley preparations market has not been accompanied by harmonized compositional standards or unified regulatory oversight. Within the European Union, such products fall under food supplement legislation, permitting only authorized nutrition and health claims supported by recognized evidence [58]. In the United States, they are regulated under the Dietary Supplement Health and Education Act (DSHEA), which allows structure–function claims but does not require a premarket demonstration of clinical efficacy [59].

A central challenge concerns the absence of universally accepted compositional standards. Unlike certain botanical extracts with pharmacopeial monographs, young barley preparations lack defined minimal concentrations of marker compounds such as saponarin or total C-glycosyl flavones. Reported concentrations of bioactive compounds vary considerably across preparations [1], reflecting differences in harvest timing, drying methods, and storage conditions [13]. Chlorophyll degradation and formation of pheophytins may occur during thermal processing [13]. Safety considerations include monitoring for heavy metals and nitrate accumulation [60,61,62]. At present, no pharmacopeial monograph exists for young barley preparations in any major pharmacopeia (European, United States, or Japanese). The establishment of analytical reference standards for saponarin and lutonarin and the development of a harmonised assay methodology would represent a critical step toward regulatory convergence and meaningful compositional benchmarking across commercial preparations.

## 7. Influence of Cultivation Practices on Phytochemical Profile and Safety

The phytochemical variability in young barley preparations arises not only from the developmental stage and processing method, but also from the agronomic environment in which the crop is grown. Nitrogen fertilization, soil composition, irrigation regime, light exposure, and crop protection strategies can all substantially modulate secondary metabolite biosynthesis and contaminant accumulation [63]. Despite the relevance of these factors, cultivation parameters are seldom reported in experimental or clinical publications.

Nitrogen availability plays a central regulatory role in plant metabolism. Moderate nutrient limitation has been associated with enhanced phenylpropanoid pathway activity and increased flavonoid accumulation in leafy crops [63,64]. Evidence from cereal systems indicates that fertilization intensity can influence the phenolic composition and antioxidant capacity [14]. Comparisons between organic and conventional cultivation systems suggest that organic production may be associated with higher concentrations of certain phenolic compounds (reported differences of approximately 19–69% for total phenolics in meta-analyses [65,66]), although the variability across species and environments is substantial [65,66]. Soil characteristics further influence mineral availability and potential heavy metal accumulation [67]. Excessive nitrogen fertilization may also increase nitrate accumulation in leafy plant tissues, a safety consideration that warrants systematic reporting in clinical studies on young barley preparations [68].

The determinants of phytochemical variability, their impact on biological activity and safety, and their implications for research standardization are summarized in Table 3. Incorporating agronomic transparency—including the reporting of cultivation system, geographic origin, fertilization regime, and contaminant screening—would markedly enhance reproducibility and translational reliability in future research.

## 8. Discussion

The body of evidence reviewed here positions young barley preparations as a complex phytochemical system with properties relevant to functional plant food applications. The co-occurrence of polyphenolic constituents, photosynthetic pigments, enzymatic antioxidants, and micronutrients supports a multi-target model of biological activity. Whether this compositional richness translates into a reproducible clinical benefit depends critically on preparation standardization, quantitative compositional characterization, and dose.

A central observation emerging from the reviewed literature is the cross-model consistency of mechanistic findings. The modulation of the redox balance, the attenuation of inflammatory signaling, and the influence on metabolic regulators constitute a coherent mechanistic framework that recurs across cellular and animal systems [31,32,33,36,37,39,40,41,42,43,44,45,51]. The translational trajectory from these experimental observations to verified clinical endpoints remains, however, incomplete.

Clinical evidence, though limited in scope and methodological rigor, provides preliminary support for the cardiometabolic potential of young barley preparations. Barley grass powder supplementation in T2DM subjects improved glycemic control and lipid profile [53], young barley leaf powder attenuated postprandial glucose increments in susceptible individuals [54], barley leaf extract reduced LDL oxidation in diabetic and hyperlipidemic cohorts [55,57], and barley green supplementation reduced serum uric acid in a randomized controlled trial [56]. The directional consistency of effects across heterogeneous preparations and endpoints—spanning glycemic control, lipid metabolism, oxidative stress, and uric acid regulation—strengthens the biological plausibility of young barley as a multi-target functional plant food.

Within the broader cereal grass category, young barley is distinguished by the presence of C-glycosyl flavones—saponarin and lutonarin—which are not reported as principal polyphenolic constituents of wheatgrass (Triticum aestivum) or other cereal grasses [1,3]. This phytochemical specificity implies that biological effects mediated through C-glycosyl flavone modulation of Nrf2 and NF-κB signaling are unlikely to be interchangeable across different cereal grass products.

A further dimension of complexity stems from agronomic variability. The nitrogen fertilization regime, soil composition, and environmental conditions can substantially reshape both the phytochemical profile and the safety characteristics of a given preparation [63,64,65,66,67,68]. This factor is seldom incorporated into clinical study design; yet, it constitutes a genuine and quantifiable source of interstudy variability.

### 8.1. Limitations

The present review is subject to several limitations. First, the absence of a systematic search protocol with pre-registered inclusion and exclusion criteria introduces the possibility of selection bias. Second, the marked variability in preparation type, extraction method, dosing regimen, and outcome measurement across primary studies precluded quantitative synthesis; all conclusions reflect a narrative rather than a meta-analytic assessment. Third, the mechanistic framework draws predominantly on in vitro data that may not translate proportionally to human biology at typical supplementation doses. Fourth, the published clinical literature on young barley preparations specifically remains limited in number, scale, and methodological rigor. Fifth, the regulatory and compositional landscape continues to evolve, and some jurisdictional details may not reflect the most current frameworks.

### 8.2. Future Research Directions

The most pressing need is methodological standardization: clinical trials should report quantitative marker compound concentrations, particularly saponarin and lutonarin, alongside the agronomic origin, processing method, and storage conditions of the preparation under investigation. Adequately powered, double-blind randomized controlled trials of sufficient duration (minimum twelve weeks) are required. Direct comparisons between barley leaf powder, grass powder, and juice-derived formulations within the same trial design would substantially clarify preparation-specific efficacy and bioavailability profiles. Dedicated human pharmacokinetic studies characterizing the absorption, distribution, and colonic biotransformation of C-glycosyl flavones are also needed. The integration of metabolomic and gut microbiome profiling into future clinical trials would facilitate identification of interindividual variability drivers and responder subgroups.

## 9. Conclusions

Young barley preparations derived from *Hordeum vulgare* L.—encompassing barley leaf, barley grass, and juice-derived products—constitute a phytochemically rich category of functional plant foods with well-documented antioxidant, anti-inflammatory, and immunomodulatory activity in experimental systems. Translated to the clinical setting, available evidence from five human intervention studies—encompassing barley grass powder, barley leaf extract, barley leaf powder, and barley green—points to potential improvements in glycemic control, lipid metabolism, oxidative stress markers, and uric acid regulation, each of which must still be regarded as preliminary.

The primary obstacle to progress is not a lack of biological activity but insufficient standardization across study levels. The explicit characterization of preparation type (barley leaf powder, barley grass powder, barley juice), routine quantification of marker compounds such as saponarin and lutonarin, and systematic disclosure of agronomic parameters are prerequisite steps toward a more robust evidence base. As an operational benchmark, a minimum saponarin content of approximately 2 mg per gram dry weight—consistent with the lower end of reported ranges in young barley preparations—could serve as a minimal compositional threshold for inclusion in future clinical trials, pending formal pharmacopeial standardization.

Rigorously designed, adequately powered randomized controlled trials employing standardized preparations and pre-specified endpoints will be necessary to determine whether young barley supplementation offers a clinically meaningful benefit within evidence-based functional nutrition.

## Figures and Tables

**Table 1:** Major bioactive compound classes identified in young barley preparations: concentration ranges, key sources of variability, stability considerations, and proposed biological relevance. DW, dry weight; SOD, superoxide dismutase; CAT, catalase; U/g, enzyme activity units per gram dry weight. Concentration ranges are indicative; enzymatic activity values refer to fresh or freeze-dried preparations.

Compound Class	Representative Compounds	Conc. Range	Key Sources of Variability	Stability	Biological Relevance	Ref.
C-glycosyl flavones	Saponarin, Lutonarin	~2–8 mg/g DW	Cultivar, harvest stage, fertilization	Relatively stable; sensitive to prolonged heat	Antioxidant; Nrf2 and NF-κB modulation	[10,11,17,18]
Phenolic acids	Ferulic, p-Coumaric, Caffeic acids	~0.1–1.5 mg/g DW	Extraction method, plant maturity, processing	Moderate; susceptible to oxidative degradation	Radical scavenging; synergistic antioxidant effects	[12,19]
Chlorophylls and derivatives	Chlorophyll a, b; Pheophytins	~3–8 mg/g DW	Growth stage, light exposure, processing	Heat-sensitive; converts to pheophytins during drying	Redox modulation; possible detoxification-related effects	[8,10,12]
Enzymatic antioxidants	SOD, Catalase	SOD: ~400–490 U/g DW; CAT: ~675–935 U/g DW	Processing method (freeze-drying vs. air-drying), storage	Highly labile; substantial loss during thermal processing	Endogenous redox support (in vitro evidence)	[13,20]
Vitamins	Vitamin C, E, β-carotene	Highly variable; dependent on drying method	Storage, oxygen exposure, processing	Vitamin C highly unstable; oxidation-sensitive	Antioxidant network support	[1,21]
Minerals	Fe, Mg, Zn, K	Soil-dependent	Soil composition, environmental exposure	Stable; may reflect soil contamination	Nutritional contribution; potential heavy metal exposure risk	[1,22]

**Table 2:** Consolidated summary of representative in vitro, in vivo, and clinical evidence for biological activity of young barley leaf, grass, and juice preparations. Studies employing barley sprout preparations were included where the investigated phytochemicals (saponarin; Nrf2/NF-κB signalling) are shared with young barley leaf and grass preparations. All in vivo and clinical studies used preparations derived from the young vegetative stage of *Hordeum vulgare* L. ROS, reactive oxygen species; NO, nitric oxide; TNF-α, tumor necrosis factor alpha; IL-6, interleukin-6; iNOS, inducible nitric oxide synthase; COX-2, cyclooxygenase-2; NF-κB, nuclear factor kappa B; Nrf2, nuclear factor erythroid 2-related factor 2; NK, natural killer cells; LDL, low-density lipoprotein; SOD, superoxide dismutase; FBS, fasting blood sugar; HbA1c, glycated haemoglobin; HDL-C, high-density lipoprotein cholesterol; LDL-C, low-density lipoprotein cholesterol; BMI, body mass index; PPAR-γ, peroxisome proliferator-activated receptor gamma.

Study Type	Model/Population	Preparation	Main Outcomes	Proposed Mechanism	Translational Relevance	Ref.
In vitro	HepG2 hepatocytes (oxidative stress)	Barley sprout extract	↓ Intracellular ROS; ↑ cell viability	Nrf2-related antioxidant signaling	Effects at supraphysiological concentrations	[31]
In vitro	LPS-stimulated RAW 264.7 macrophages	Saponarin-containing barley extract	↓ NO; ↓ TNF-α and IL-6	NF-κB inhibition; ↓ iNOS and COX-2	Isolated compound effects may not reflect whole preparation	[33]
In vitro	Human cancer cell lines (HT-29 colon, A549 lung, breast)	Water and juice extracts of young barley grass	↓ Cell proliferation; pro-apoptotic signaling	Mitochondrial membrane depolarization; caspase activation	Concentration-dependent; potential functional food application	[41,42,43,44]
In vitro	NK-92/LS180 colon cancer cells	Polysaccharide-rich young barley water extract	↑ NK cell cytotoxicity against cancer cells	Immunomodulatory activity of barley fructooligosaccharides	Novel immunological mechanism; relevant to functional food context	[45]
In vitro	Human dermal fibroblasts (UV-induced oxidative injury)	Young barley grass dietary supplement extract	↓ Lipid peroxidation; ↑ antioxidant enzyme activity	Redox modulation; enhanced cellular antioxidant defense	Relevant to physiological oxidative stress protection	[9]
In vivo	Dietarily induced hypercholesterolaemia (rats)	Green juice from young barley leaves	↓ Total cholesterol; ↓ LDL; improved lipid profile	Antioxidant and hypolipidaemic effects of barley leaf constituents	Early foundational study; preparation not fully characterized	[51]
In vivo	High-fat diet atherosclerosis model (rabbits)	Barley leaf essence	↓ Blood cholesterol; ↓ triglycerides; ↓ atherosclerotic markers	Antioxidative and hypolipidaemic activity of barley leaf phytochemicals	Rabbit model; comparable to human cardiovascular disease context	[39]
In vivo	High-fat diet-induced obesity (rats)	Barley grass juice (*Hordeum vulgare* L.)	↓ Body weight; ↓ BMI; improved lipid profile; ↓ hepatic markers	PPAR-γ and caspase-3 modulation in liver	Barley grass juice preparation; 60-day intervention	[40]
Clinical	Type 2 diabetes mellitus (adults, n = 59)	Barley grass powder (BGP, 1.2 g/day, 60 days)	↓ FBS; ↓ HbA1c; ↓ total cholesterol; ↓ LDL-C; ↑ HDL-C	Multi-target metabolic modulation by barley grass bioactives	Non-randomized; no blinding; small sample; short duration	[53]
Clinical	Healthy volunteers and high-postprandial glucose subjects	Young barley leaf powder (BLP, 1.8 g/serving)	↓ Postprandial blood glucose in high-glucose responders	Increased digesta viscosity by insoluble fiber from barley leaf	Effect limited to subjects with higher postprandial glucose	[54]
Clinical	Type 2 diabetes (adults); barley leaf extract + antioxidant vitamins	Young barley leaf extract supplement	↓ LDL oxidation; ↑ free radical scavenging activity	Antioxidant activity of C-glycosyl flavones and phenolic acids	Combined intervention; limited duration; oxidative stress outcomes only	[55]
Clinical	Adults with hyperuricemia (n = 90)	Barley green (young barley grass), 3 months	↓ Serum uric acid; ↓ XOD activity; improved body composition	Inhibition of xanthine oxidase activity by barley-derived flavonoids	RCT; most methodologically rigorous trial to date; novel endpoint (uric acid)	[56]
Clinical	Hyperlipidemic smokers and non-smokers (n = 40)	Young barley leaf extract (15 g/day, 4 weeks)	↓ Total cholesterol; ↓ LDL-C; ↑ lag phase of LDL oxidation	Antioxidant activity of C-glycosyl flavones; inhibition of LDL oxidation	No placebo control; stronger antioxidative effect in non-smokers	[57]

**Table 3:** Determinants influencing phytochemical variability, bioactivity, and safety profile of young barley preparations.

Determinant Category	Specific Factor	Impact on Phytochemical Profile	Impact on Safety	Research Implication	Ref.
Agronomic	Nitrogen fertilization intensity	Modulates phenylpropanoid pathway; alters C-glycosyl flavone concentration	May increase nitrate accumulation in leafy tissues	Fertilization regime should be reported in all studies	[14,63,64]
Agronomic	Organic vs. conventional cultivation	Potential variation in total phenolic content; evidence inconsistent across species	Affects likelihood of synthetic pesticide residues	Compositional verification required; label-based assumptions insufficient	[65,66]
Environmental	Soil composition and mineral availability	Influences micronutrient and trace element content	Primary determinant of heavy metal exposure risk	Soil origin and contaminant testing should be documented	[67]
Harvest	Growth stage at harvest	Alters chlorophyll and flavone concentration; peak at early vegetative stage	Minimal direct safety effect	Precise harvest timing (e.g., days post-germination) should be specified	[10,12]
Processing	Drying method (air-drying vs. freeze-drying)	Affects vitamin C, enzymatic activity, and flavonoid stability	Indirect via formation of degradation products	Processing method and temperature must be clearly described	[7,13]
Post-processing	Storage temperature and humidity	Influences degradation kinetics of phenolics and chlorophyll derivatives	May affect microbial stability	Stability data and storage conditions should accompany clinical trial reporting	[13]

## Data Availability

No new data were created or analyzed in this study. Data sharing is not applicable to this article.
